# Comparison of Foam Glass-Ceramics with Different Composition Derived from Ark Clamshell (ACS) and Soda Lime Silica (SLS) Glass Bottles Sintered at Various Temperatures

**DOI:** 10.3390/ma14030570

**Published:** 2021-01-26

**Authors:** Noor Aizat Noor Hisham, Mohd Hafiz Mohd Zaid, Sidek Hj Ab Aziz, Farah Diana Muhammad

**Affiliations:** 1 Department of Physics, Faculty of Science, University Putra Malaysia, Serdang 43400, Selangor, Malaysia; aizatizme95@gmail.com (N.A.N.H.); sidek@upm.edu.my (S.H.A.A.); farahdiana@upm.edu.my (F.D.M.); 2 Materials Synthesis and Characterization Laboratory, Institute of Advanced Technology, University Putra Malaysia, Serdang 43400, Selangor, Malaysia

**Keywords:** soda lime silica glass, ark clamshell, foam glass-ceramics, different composition, wollastonite, compressive strength, porosity

## Abstract

Soda lime silica (SLS) waste as the source of silica (SiO_2_) and ark clamshell (ACS) as the foaming agent has been utilized to fabricate the low-cost and lightweight foam glass-ceramics. A series of 1 and 6 wt% foam glass-ceramics were successfully prepared by the conventional solid-state sintering method at various sintering temperatures for 60 min. The bulk density of the samples has achieved minimum density (1.014 g/cm^3^) with maximum expansion (62.31%) at 6 wt% of the ACS content sintered at 800 °C for 60 min. The bulk density increases while the linear shrinkage and total porosity decrease with the progression of ACS contents and sintering temperature, where the results correspond with the FESEM micrograph. The result of XRD and FTIR transmittance spectra have shown that the formation of wollastonite crystal has occurred starting at 6 wt% of the ACS content sintered at 800 °C for 30 min. The highest mechanical performance (3.90 MPa) with an average total porosity (8.04%) is observed for the sample containing 1 wt% of ACS. It can be concluded that the composition of foam glass-ceramics (1 and 6 wt%) and sintering temperatures give significant results to the structural, physical, and mechanical properties of the fabricated foam glass-ceramics.

## 1. Introduction

Foam glass-ceramics are porous materials that are typically formed before undergoing the sintering process by mixing carbonaceous substances with the glass. Some materials generally have a porosity over 60% of the total volume, which can be closed, opened, or both [1]. Foam glass-ceramics show remarkable mechanical performance and have attracted considerable interest from the construction industry due to features such as lightweight, rigidity, and high compressive strength, as well as being chemically inert and non-toxic [2]. These are, therefore, commonly used as building blocks for the protection of roofs, walls, floors, and ceilings at high and low temperatures, as fillers for the reconstruction of collapsed sloops, as sub-aggregates for the improvement of structures, as lightweight aggregates for concrete composition and water folding material for greening [3]. Such materials can also be used as scaffolds for the engineering of bone tissue [4,5].

Soda lime silica (SLS) glass is the most common form of commercial silicate glass and the least expensive [6]. This glass is mainly used for windows because the glass can transmit a high percentage of visible light [7]. Additionally, it is also used in glass containers as it is virtually inert and non-toxic [8,9,10]. Other than that, SLS glasses have possibilities and advantages to be applied in medical applications such as bioactive glasses [11]. SLS glass contains about 70–75% silica (SiO_2_), 12–15% sodium oxide (Na_2_O), and 10–15% calcium oxide (CaO) with other minor oxide composition such as potassium oxide (K_2_O) and aluminum oxide (Al_2_O_3_) [12]. The glass cullet has been known for its ability to provide an interesting property in ceramics-based products [1]. Therefore, they are used in the fabrication of foam glass-ceramics as an alternative way of processing glass waste and an ideal candidate as a natural resource replacement.

Introducing a foaming agent or pore-forming agent into the ceramic matrix is the most common technique in the manufacturing process of foam glass-ceramics [1]. Foaming agents, which are also known as blowing or expanding elements, are the compound introduced into the matrix to extend the cross-linked arrangement and form a porous structure under suitable conditions [13]. The foaming ability is usually depending on the decomposition process of the carbonates or sulfates [14]. In glass-ceramics applications, inorganic foaming agents have been used in previous research, such as C, AlN, CaCO_3_, MnO_2_, and SiC, which have presented the degree of success of the work [15,16,17,18,19,20]. Recently, various studies have been conducted by using waste products from seafood waste containing carbonates as a foaming agent, including ark clamshell (ACS). ACS can provide many additional benefits and can be a low-cost alternative as a foaming agent [21].

ACS, also known as mollusk shell, is a clam or bivalve mollusk in the family of Veneridae [22]. In certain countries, ACS is available in large amounts and is typically discarded in landfills. Global production of bivalve mollusks has increased dramatically over the last 60 years, from almost 1 million tonnes in 1950 to about 14.6 million tonnes in 2015 [23]. ACS consists mainly of CaCO_3_ at 95–99% and with other minor oxides such as potassium (K), silicon (Si), iron (Fe), and strontium (Sr) [24]. A comparison of the measurement of XRD between one type of ACS (*Anadara granosa*) and one of mussel shell (MS) (*Perna viridis*) can be seen in the work of Siriprom et al. (2012). The composition of the ACS is 100% aragonite (CaCO_3_) while that of the MS is 13% calcite (CaCO_3_) and 78% aragonite (CaCO_3_) [25].

In this study, a series of foam glass-ceramics were prepared by a controlled sintering process using the powder method and a single-stage conventional solid-state method. The foam glass-ceramics properties were characterized by physical structural, chemical, morphological, and mechanical behaviors to investigate the effect of the ACS composition with the progression of the sintering temperature for the formation of foam glass-ceramics. The physical, structural, and mechanical properties of foam glass-ceramics had been studied through measurements such as density, linear shrinkage, total porosity, X-ray diffraction (XRD), Fourier transform infrared (FTIR), field emission scanning electron microscope (FESEM), and compressive strength using a universal test machine (UTM). The main purpose of this work is to fabricate foam glass-ceramics from waste materials as a potential concrete material use in the field of building and construction.

## 2. Materials and Methods

### 2.1. Sample Preparation

Waste SLS glass and ACS were collected from recycling sites at Taman Sri Serdang, Sri Kembangan, Selangor, Malaysia. Both of these raw materials were first cleaned and dried to prevent any contamination. Then, the raw materials were smashed into small pieces using plungers. The raw materials were then milled separately for 24 h at 50 rpm to obtain finer powders by using a porcelain milling jar containing alumina balls. The resulting fine powders were ground with an agate mortar to obtain a finer powder before it was sieved to the desired particle size fraction of ≤45 µm. The chemical composition of the SLS glass and ACS can be found in Table 1.

Both of the powders were then placed on weighing paper and weighed approximately 1 g using a digital weighing balance (A&D, Micro Analytical Weighing, GR-200, Tokyo, Japan) according to the desired composition based on the empirical formula as shown in Table 1. The powders that were weighed according to the composition were homogenously mixed by using a pestle and mortar before they were ready to go through the pelleting process by using a manual hydraulic press with an applied load of five tons. The powders were bound with two drops of polyvinyl alcohol (PVA) and pressed into a pellet with a diameter of ~13 mm and a thickness of ~2 mm. Subsequently, the resulting pellet was left for a day before being subjected to the sintering process. The samples were then sintered at various temperatures of 700, 800, and 900 °C for 60 min in a conventional chamber furnace with a heating rate of 10 °C/min in atmospheric ambient conditions. Each of the samples (1 and 6 wt%) was subjected to the sintering process one time for each sintering temperature. The sintering process yielded 14 foam glass-ceramics samples. Finally, some of the foam glass-ceramics samples were manually crushed into powder form and sieved beforehand at a particle size fraction of 45 µm for characterization process.

### 2.2. Materials Characterizations

The chemical composition of the raw materials was analyzed by using a wavelength dispersive X-ray fluorescence (WDXRF) spectrometer (Bruker, model S6 Jaguar, Billerica, Madison, WI, USA). Meanwhile, thermogravimetric analysis (TGA) (Mettler Toledo, model TGA/DSC 1 HT, Columbus, OH, USA) was used with a flow rate of about 50 cm^3^/min of nitrogen gas to study the properties and thermal behavior of ACS. The material was prepared in a powder form with a particle size of 45 µm. The crystalline phase of the raw materials and foam glass-ceramics of different composition was identified using powder X-ray diffraction (XRD). This XRD measurement was performed using an PANalytical X’PERT PRO-MPD diffractometer (Malvern PANalytical, Philips PW3040/60 model, Malvern, UK) using Cu Kα radiation of 40 kV with an input current of 30 mA in a 2θ range from 20 to 80°. Methods for analyzing crystalline phases were performed using X’PERT HighScore Plus version 3.0 software. Subsequently, foam glass-ceramics samples were subjected to Fourier transform infrared reflection spectroscopy (FTIR) (Thermo Scientific, Nicolet 6700 model, Waltham, MA, USA), to identify functional groups contained in the sample to support the results of XRD characterization in the wavenumber within the range of 400–4000 cm^−1^. For morphological characterization, FESEM (FEI, NOVA NanoSEM 230 model, Hillsboro, OR, USA) was used to see and distinguish porous walls and pore structures of the samples.

For physical properties, bulk density, *ρ_b_* for the sintered sample was obtained using the Archimedes principle based on the following formula:

(1) $$\rho_{b}=\frac{W_{air}}{W_{air}-W_{distilled\;water}}W_{distilled\;water}$$

where the distilled water density is 1.0 g/cm^3^. The theoretical density, *ρ_t_* measurements were performed using the Brunauer-Emmett-Teller instrument, BET (Micromeritics Gemini 2375 model, Georgia, GA, USA). The total porosity, *ε* of the foam glass-ceramics was also estimated using the bulk density, *ρ_b_*, and theoretical density, *ρ_t_*, with the results according to the following formula:

(2) $$\mathrm{Total}\;\mathrm{porosity},\;\left(\varepsilon \right)=\left(1-\frac{bulk\;density}{theoretical\;density}\right)\times 100\;\%$$

Additionally, linear shrinkage, *LS* of foam glass-ceramics was also obtained by measuring the sample diameter before and after the sintering process using a digital Vernier caliper (Mitotoyo, ± 0.01 mm). The data for the measured samples were taken and the linear shrinkage, *LS* percentages were manually calculated according to the following formula:

(3) $$\mathrm{Linear}\;\mathrm{Shrinkage},\;\left(LS\right)=\;\frac{Initial\;diameter-Final\;diameter}{Initial\;diameter}\times 100\%$$

The samples were measured and repeated five times and the average value of the linear shrinkage, *LS* obtained from the calculation was recorded.

Characterization for determining the mechanical properties of the foam glass-ceramics sample was performed by compressive strength measurement using a universal hydraulic press (EMIC-DL 2000 model) with 10 mm/min crosshead speed with 10 KN load. Foam glass-ceramic samples were cut to ensure that both the top and bottom surfaces of the sample were flat and perpendicular using a low-speed precision cutting machine (Buehler, IsometTM model, Lake Bluff, IL, USA). The procedure was done to standardize the sample in a disc size of approximately 4.0 × 8.0 mm^2^. It is worth mentioning that a thin sponge layer covered the loaded surfaces for the sample faces to obtain uniform load distribution. There were five samples used for each measurement of the compressive strength and the average data were recorded. These characterization results were measured from the stress-strain curve at the first maximum of the highest strength point.

## 3. Results and Discussion

### 3.1. Raw Materials Analysis

The chemical composition of SLS and ACS raw materials was analyzed using wavelength dispersive X-ray fluorescence (WDXRF). The chemical analysis result is shown in Table 2 and all elements had been measured in the form of oxides. As can be seen in Table 2, the major components of SLS are SiO_2_, CaO, and Na_2_O where these oxide elements consist of 96.59 wt% of the total weight of the SLS composition. Other oxide elements, such as Al_2_O_3_, CaO, and MgO, are minor constituents contained in the SLS composition. Meanwhile, the major components of oxide elements found in ACS composition are only CaO while the other oxides element such as Na_2_O and MgO are minor constituents. Other oxide elements in ACS are mostly lost in ignition (LOI) and collectively amounted to 45.23 wt%.

**Figure 1:**
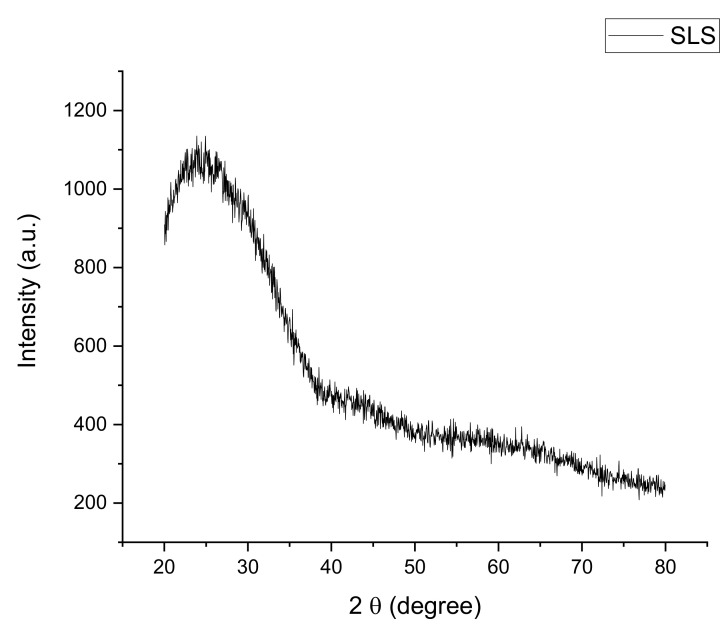
The XRD pattern of waste SLS glass powder at room temperature.

**Figure 2:**
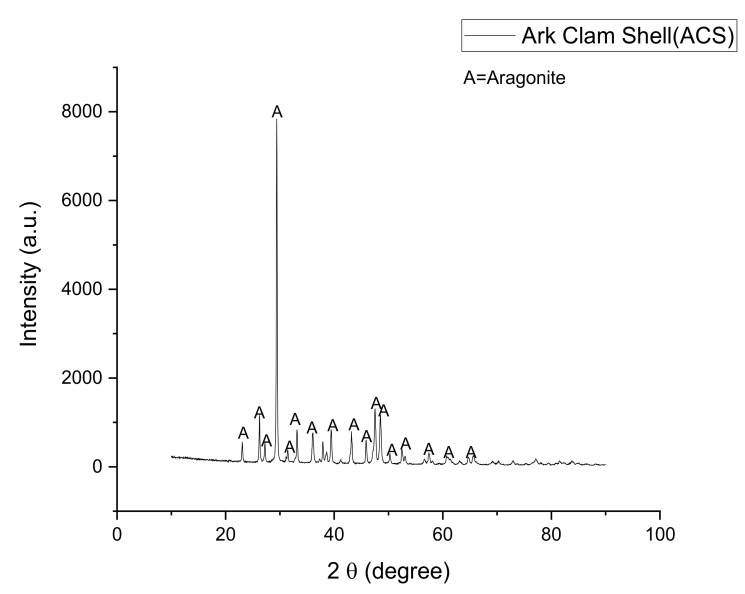
The XRD pattern of waste ACS powder at room temperature.

XRD patterns of SLS glass and ACS powder at room temperature are shown in Figure 1 and Figure 2, respectively. Based on Figure 1, broad halos at an angle around 20 to 40° are observed on the SLS glass powder sample. The SLS glass powder XRD pattern reveals no continuous or discrete sharp peak. Nevertheless, the existence of a broad feature of the amorphous halo can be seen with a short-range structural disorder that represents the SLS glass’ amorphous nature.

Based on Figure 2, the shape of the peak for the ACS powder sample signifies the reflection of the aragonite crystalline phase (JCPDS no. 96-900-0227). Aragonite crystal phase is a mineral carbonate with an orthorhombic crystal system which is the most common form of CaCO_3_ naturally occurring crystal [26,27]. Raw materials from a waste product with relatively high CaCO_3_ content are needed in this study to promote the foaming process in producing samples of foam glass-ceramics [28].

**Figure 3:**
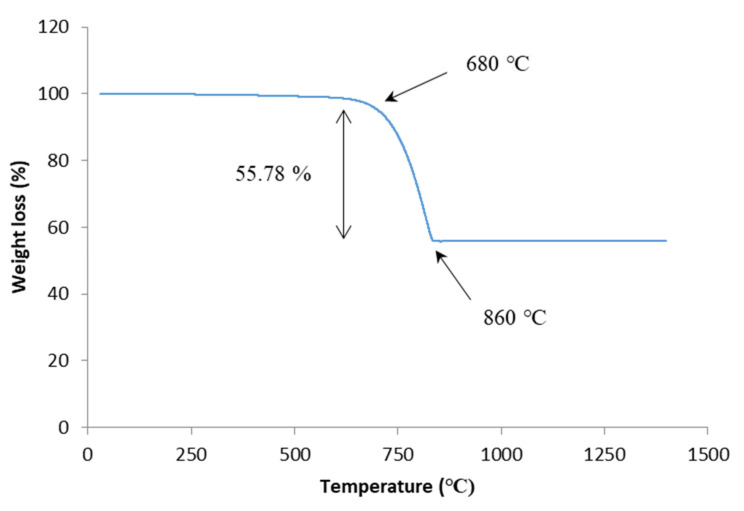
The TGA result of ACS weight loss against temperature.

Figure 3 displays the results of the thermogravimetric analysis of the ACS powder which was used as a foaming agent to fabricate the foam glass-ceramics samples. The thermal properties of ACS indicate that the gradual decomposition of CaCO_3_ has occurred after undergoing the sintering process around 800 °C, which is caused by a mass loss of approximately 55.78% during the sintering process at temperatures around 700 °C [29]. At a higher temperature of 900 °C, it is observed that no change has occurred in the CaCO_3_ decomposition process. This results in no further expansion of the foam glass-ceramics samples [30].

### 3.2. Foam Glass-Ceramics Analysis

#### 3.2.1. Structural Studies

**Figure 4:**
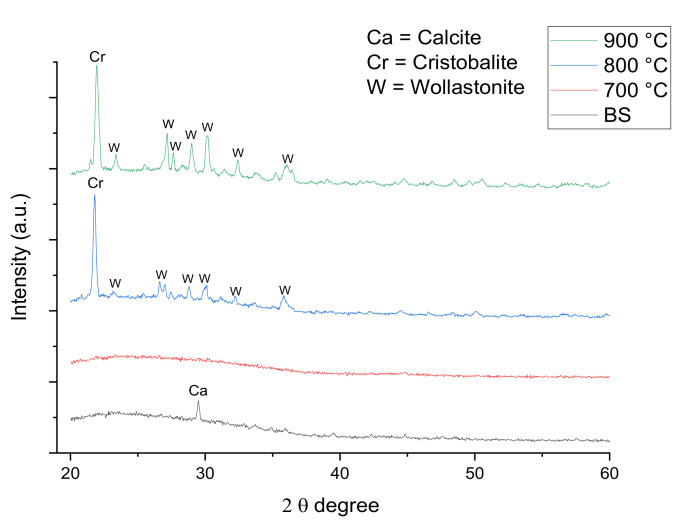
XRD pattern of foam glass-ceramics 1 wt% sintered at various temperatures for 60 min.

**Figure 5:**
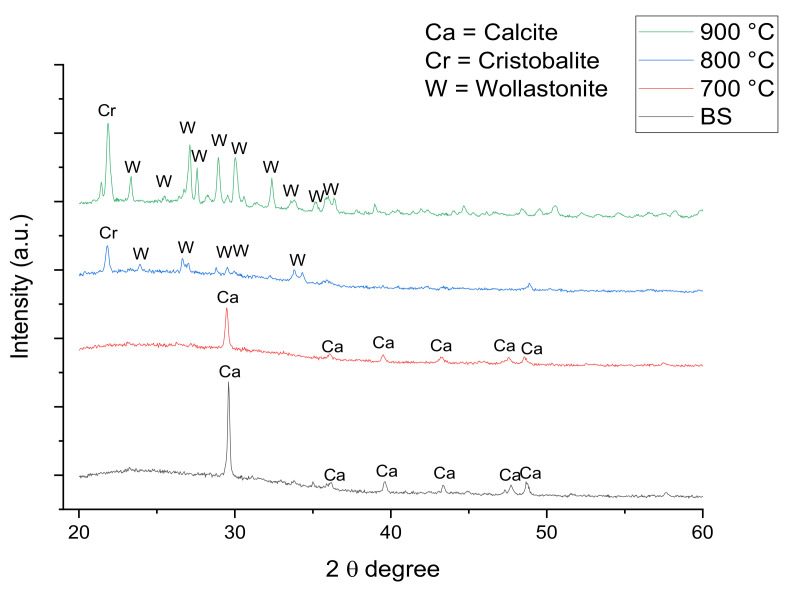
XRD pattern of foam glass-ceramics 6 wt% sintered at various temperatures for 60 min.

The XRD pattern of the samples of foam glass-ceramics with 1 and 6 wt% of ACS sintered at various temperatures for 60 min are shown in Figure 4 and Figure 5, respectively. The results depicted in Figure 4 and Figure 5 indicate that the sample before sintering (BS) has small peak that belongs to the calcite crystalline phase (CaCO_3_, JCPDS: 96-901-6707) at the peak around 2θ = 29.5° for 1 wt% sample while 6 wt% sample have additional calcite peaks around 2θ = 6.1, 39.6, 43.4, 47.7, and 48.7°. The calcite crystal phase starts to disappear at the XRD pattern of 1 wt% sintered at 700 °C for 60 min as can be seen in Figure 4. This is due to the decomposition of CaCO_3_ during the sintering process. On the other hand, the calcite crystals phase for 6 wt% samples sintered at 700 °C for 60 min are still present. This is because the ACS content in the sample is high and the CaCO_3_ have not been completely decomposed due to insufficient holding time.

As the sintering temperature increases to 800 °C, a minor reflection of a cristobalite crystal phase (SiO_2_, JCPDS: 96-900-8229) with a tetragonal crystal system and major reflection of a wollastonite crystal phase (CaSiO_3_, JCPDS: 96-900-5778) with a monoclinic crystal system is detected. The cristobalite crystal peak has started to appear around 2θ = 21.7° for both 1 and 6 wt% of samples. This happens due to the reaction of SiO_2_ that begins to bond with the CaO at a high sintering temperature [31]. However, the intensity of the cristobalite crystal peak for 6 wt% sample sintered 800 °C is lower when compared to the intensity of the cristobalite crystal peak for 1 wt% sintered 800 °C samples. Such observation is due to the higher content of CaO in 6 wt% samples sintered 800 °C. The wollastonite crystal peaks for 1 wt% sample sintered at 800 °C are observed to appear around 2θ = 23.9, 26.7, 28.7, 29.4, 33.7, and 35.8°. For 6 wt% sample sintered at 800 °C, the wollastonite crystal peaks are observed around 2θ = 23.9, 26.7, 28.7, 29.4, and 34.3° [32].

The intensity of wollastonite crystal peaks at 6 wt% sintered at 800 °C are low compared to the 1 wt% sample sintered at 800 °C. By increasing the sintering temperature up to 900 °C, the intensity of cristobalite peaks for both 1 and 6 wt% are found to increase and the intensity of wollastonite crystal peaks has become more intense with the presence of new sharp peaks. From a previous study, it is reported that the cristobalite crystal structure tends to grow at high sintering temperatures [33]. However, the intensity of the cristobalite crystal peak is lower when compared to that of the composition from 1 to 6 wt%. This is because more CaO in 6 wt% sample has bonded with SiO_2_. The wollastonite crystal phase intensity is intense at a 900 °C sintering temperature since the CaCO_3_ has become saturated at 860 °C based on the TGA result in Figure 3.

**Figure 6:**
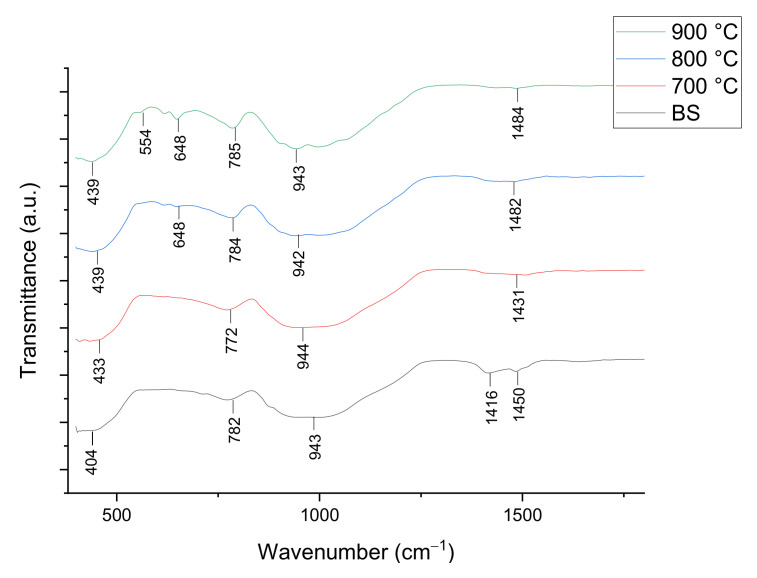
FTIR spectra of foam glass-ceramics 1 wt% sintered at various temperatures for 60 min.

**Figure 7:**
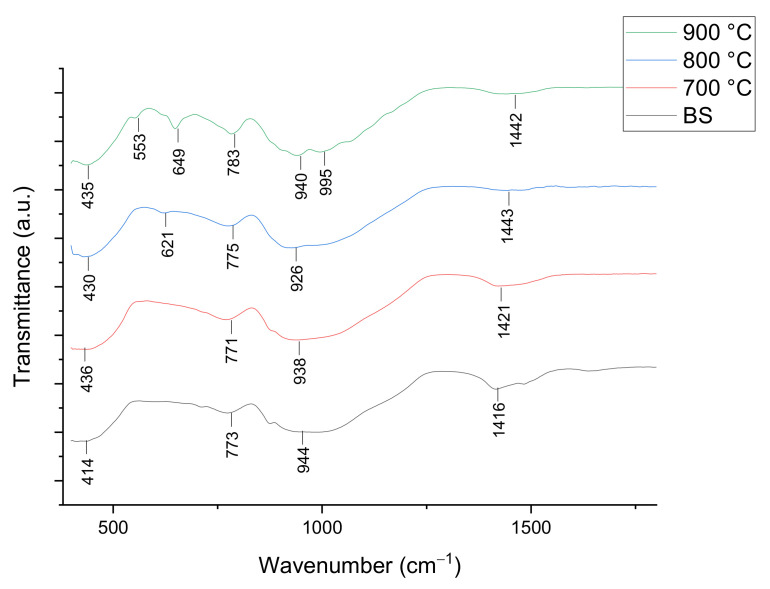
FTIR spectra of foam glass-ceramics 6 wt% sintered at various temperatures for 60 min.

The FTIR spectra of 1 and 6 wt% samples sintered at 800 °C for 60 min are shown in Figure 6 and Figure 7, respectively. The IR absorption band and foam glass-ceramics band assignment for 1 and 6 wt% samples at various temperatures can be seen in Table 3. Based on the figure shown, there are intense peaks of C–O bond at the wavenumber of 1416 and 1450 cm^−1^ for the 1 wt% sample and 1416 cm^−1^ for the 6 wt% samples before sintering (BS). Other than that, the main strong peaks are observed at 943 and 782 cm^−1^ for the 1 wt% sample and 944 and 773 cm^−1^ for the 6 wt% sample, corresponding to the asymmetric stretching and symmetric stretching of O–Si–O bond respectively. Additionally, the peaks at 648 and 649 cm^−1^ support the progress of wollastonite crystal growth with the existence of a Ca-O-Si vibration band at a sintering temperature above 800 °C.

The wavenumber of 404 cm^−1^ for the 1 wt% sample and 414 cm^−1^ for 6 wt% belong to the O-Si-O bending mode. After the sintering temperature increases from 700 to 900 °C, the intensity of C-O has weakened due to the decomposition of CaCO_3_ by the CO_2_ gas releasement [34]. Apart from that, the intensity of O–Si–O is observed to decrease slightly when sintered from 700 to 800 °C for the samples of both compositions. Meanwhile, the intensity of the O-Si-O vibration band at 900 °C sintering temperature has increased slightly compared to 800 °C sintering temperature. Additionally, the vibration band at 648 cm^−1^ for the 1 wt% sample and 621 cm^−1^ for the 6 wt% samples that appear at 800 °C does not exist after the sintering process at 700 °C. The vibration bands are only visible at 800 °C and later become more intense at a 900 °C sintering temperature, which is due to the presence of the wollastonite crystal phase at high sintering temperature.

**Figure 8:**
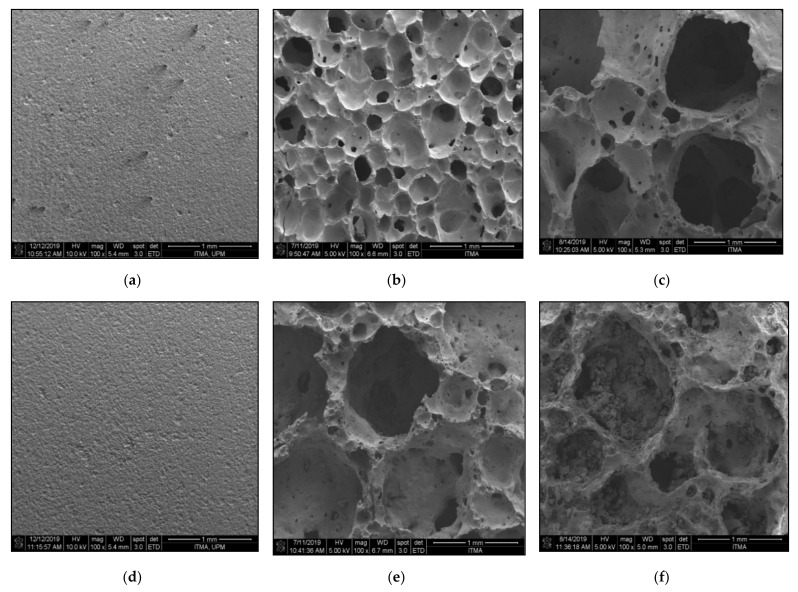
FESEM micrograph of foam glass-ceramics sintered for 60 min at various temperature (**a**) 1 wt% 700 °C, (**b**) 1 wt% 800 °C, (**c**) 1 wt% 900 °C, (**d**) 6 wt% 700 °C, (**e**) 6 wt% 800 °C, (**f**) 6 wt% 900 °C.

Figure 8 shows the FESEM micrograph of the foam glass-ceramics samples in parallel cross-section sintered at various temperature for 60 min. The results are used to visualize the microstructure details on the surface crystallization of the samples. As can be seen in Figure 8a,d), there are no obvious differences that can be observed. The pores’ structures are lacking in the cross-section of the sample’s microstructures at 1 and 6 wt% samples sintered at 700 °C. This is because the gas generated by the foaming agent at 700 °C gives insufficient pressure to cause the expansion on the glass matrix. When the sintering temperature increases to 800 °C, the pores structure begins to become larger and well foamed for 6 wt% samples as shown in Figure 8e). The FESEM micrograph reveals that the 6 wt% sample has the largest pores structure with the thickest pore walls at 800 °C. However, Figure 8b) shows that 1 wt% sample sintered at 800 °C has the smaller pores with homogenous pore’s distribution compared to 6 wt% samples sintered at the same sintering temperature. As the temperature increases to 900 °C, pores with large diameter start to form on the 1 wt% sample microstructure. The pore walls have also become thicker as can be seen in Figure 8c) compared to the microstructure in Figure 8b). However, the 6 wt% samples are found to undergo deformation, with more densified pore walls compared to the 6 wt% samples sintered at 800 °C, as shown in Figure 8f).

#### 3.2.2. Physical Studies

**Figure 9:**
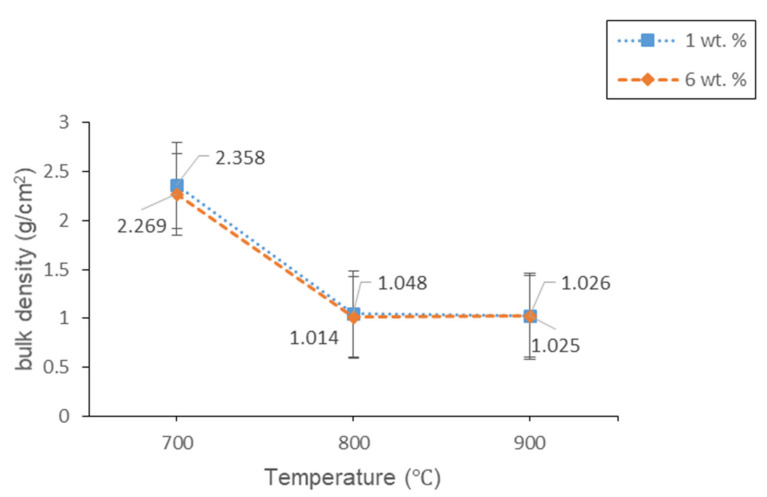
The bulk density of 1 and 6 wt% samples sintered at various temperatures for 60 min.

**Figure 10:**
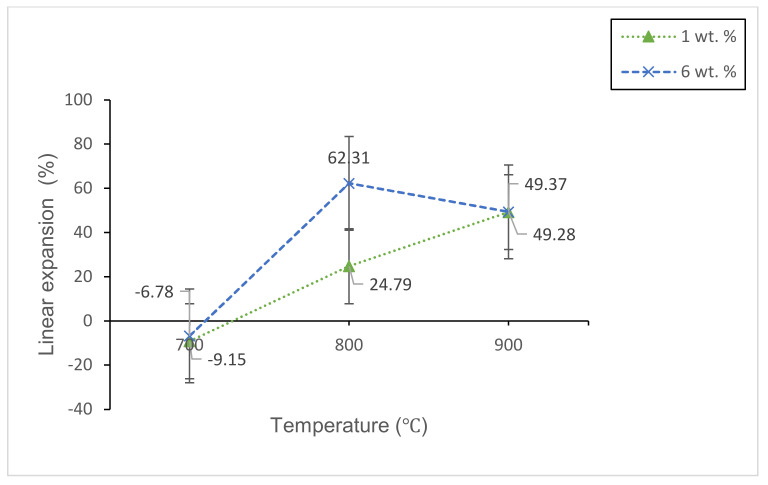
The linear shrinkage of 1 and 6 wt% samples sintered at various temperatures for 60 min.

Figure 9 and Figure 10 show the bulk density and linear shrinkage of 1 and 6 wt% samples sintered at various sintering temperature for 60 min, respectively. The lowest bulk density (1.014 g/cm^3^) with maximum expansion (62.31%) is achieved in 6 wt% samples sintered at 800 °C. While the highest bulk density (2.358 g/cm^3^) with the negative linear shrinkage (−9.15%) is found in 1 wt% sample sintered at 700 °C. Such a result is obtained due to insufficient energy of the sample for the optimum foaming process. The glass densification that happens at 700 °C results in the shrinkage of the samples. Based on Figure 9 and Figure 10, both of the samples have a high bulk density value at 700 °C. Meanwhile, when the temperature increases to 800 °C, the bulk density value for both samples decrease and the linear shrinkage increases. This result shows that the CO_2_ gas released by the foaming agent has partially been retained on the glass matrix, which causes its expansion and the formation of pores [19]. The formation of pores on the glass matrix has resulted in low bulk density and high expansion of the samples. The high viscosity of the molten glass when sintered at 800 °C also causes the glass to be relatively dense and promotes the expansion and pore formation of the samples [35]. By increasing the sintering temperature up to 900 °C, the bulk density of 6 wt% samples is found to increase and the linear shrinkage is found to decrease. While the bulk density for 1 wt% of the sample continues to decrease and the linear shrinkage continues to increase. This happens because the 6 wt% sample has more foaming agent content compared to 1 wt% sample. The high energy involved during the sintering process at high temperatures has resulted in an intense gas flow on the glass matrix. The intense gas flow ruptures the pore walls inside the glass matrix of 6 wt%, which leads to an increase in the bulk density as well as a decrease in linear shrinkage. From the results, it can be seen that the bulk density has an inverse relationship with that of the linear shrinkage results.

**Figure 11:**
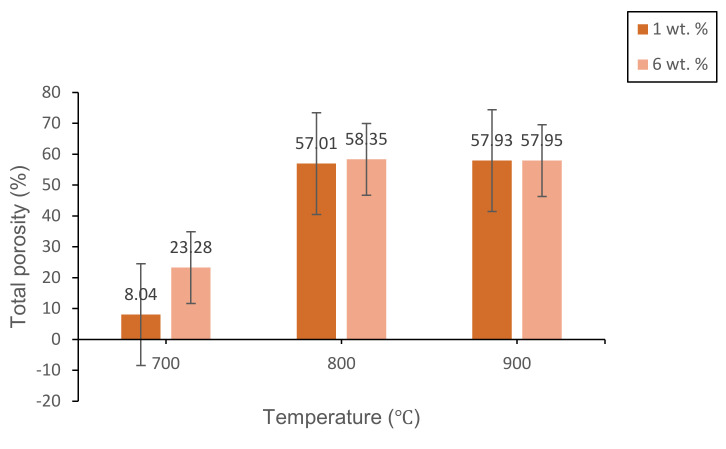
The total porosity of 1 and 6 wt% samples sintered at various temperatures for 60 min.

Figure 11 shows the total porosity result of 1 and 6 wt% samples sintered at various temperatures for 60 min. The result has a linear relationship with respect to the trend of bulk density result and FESEM micrograph. This is because the density of foam glass-ceramics is associated with the pore formation inside the glass matrix. Based on the results obtained, the maximum total porosity (58.35%) is achieved at 6 wt% samples sintered at 800 °C while the minimum total porosity (8.04%) is achieved at 1 wt% sample sintered at 700 °C. The results are in good agreement with the FESEM micrograph in Figure 8a,c. Overall, the result in Figure 8c has a well-formed large pore distribution which reveals up to 58.35% of total porosity. Meanwhile, the FESEM micrograph in Figure 8a has the homogenous distribution of pores with a small diameter and has a total porosity of 8.04%.

#### 3.2.3. Mechanical Studies

**Figure 12:**
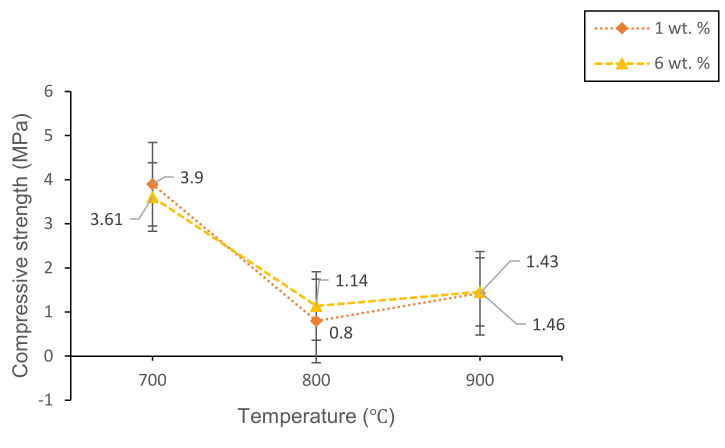
The compressive strength of 1 and 6 wt% samples sintered at various temperatures for 60 min.

Figure 12 shows the compressive strength of 1 and 6 wt% samples sintered at various temperatures for 60 min. The highest compressive strength (3.90 MPa) is achieved at 1 wt% sample sintered at 700 °C while the lowest compressive strength (0.80 MPa) is achieved at 1 wt% sintered at 800 °C. At 700 °C, the 1 wt% sample shows a greater compressive strength result compared to the 6 wt% sample. This is because the bulk density (2.269 g/cm^3^) and total porosity (8.04%) of 6 wt% sample are lower than the bulk density (2.358 g/cm^3^) and total porosity (23.28%) of 1 wt% sample, respectively. When the sintering temperature increases to 800 °C, the compressive strength of both 1 wt% and 6 wt% become lower compared to the samples sintered at 700 °C. This is because the pore foaming process results in the distribution of larger pores due to the CO_2_ gas releasement during the sintering process. The pore structure formed on the glass matrix gives an adverse effect on the mechanical properties of foam glass-ceramics [36]. However, the compressive strength (1.14 MPa) of the 6 wt% sample is greater than the compressive strength of the 1 wt% sample. The result is probably due to higher CaO content in 6 wt% sample, which results in a higher intensity of the wollastonite crystal phase formed within the sample. As the sintering temperature reaches 900 °C, the compressive strength for both 1 and 6 wt% samples increase. The compressive strength of the 6 wt% sample is greater than that of the 1 wt% due to the ruptured pore walls and affect further densification as can be seen in Figure 8f.

## 4. Conclusions

Lightweight foam glass-ceramics with different compositions (1 and 6 wt%) were successfully fabricated via a solid-state sintering method at various temperatures for 60 min. ACS possesses high percentages of CaCO_3_ (100% aragonite) and has been used as the foaming agent in this study. The different percentages of ACS content and sintering temperature play an important role in determining the structural, physical, and mechanical properties of the foam glass-ceramics. The wollastonite (CaSiO_3_) crystal phase is detected at 800 °C for both samples. The crystal peaks of wollastonite continue to grow at high sintering temperatures and become more intense with the appearance of new sharp peaks at 900 °C for 6 wt% samples. Meanwhile, the FTIR spectra reveal the vibration band at 648 cm^−1^ for 1 wt% sample and 621 cm^−1^ for 6 wt% samples sintered at 800 °C, which correspond to the formation of wollastonite crystal phase. The vibration band slightly increases at 900 °C due to the presence of wollastonite crystal phase at high sintering temperature, which supports the result of XRD analysis. As for the microstructure of foam glass-ceramics, the FESEM micrograph has revealed that the 6 wt% sample sintered at 800 °C shows the largest pores diameter with the thickest pore walls. Other than that, the lowest bulk density (1.014 g/cm^3^) with maximum expansion (62.31%) is achieved at 6 wt% samples sintered at 800 °C, while the highest bulk density (2.358 g/cm^3^) with the negative linear shrinkage (−9.15%) is found at 1 wt% sample sintered at 700 °C. Additionally, the maximum total porosity (58.35%) is achieved in 6 wt% samples sintered at 800 °C while the minimum total porosity (8.04%) is achieved in 1 wt% sample sintered at 700 °C. The total porosity results are in good agreement with the FESEM micrograph. Lastly, the highest compressive strength (3.90 MPa) is acquired in 1 wt% sample sintered at 700 °C while the lowest compressive strength (0.80 MPa) is achieved in 1 wt% sintered at 800 °C. Samples with 6 wt% ACS sintered at 800 °C could be potentially used as a concrete material in the field of building and construction.

## Figures and Tables

**Table 1:** Ratios of ACS to waste SLS glass powder.

Empirical Formula [ACS]*_x_*[SLS]_100−*x*_	Weight (g)		
	ACS	SLS	ACS-SLS
1(ACS)99(SLS)	0.01	0.99	1.00
6(ACS)94(SLS)	0.06	0.94	1.00

**Table 2:** Chemical composition of SLS glass and ACS.

Oxides (wt%)	Raw Materials	
	SLS ± 0.1	ACS ± 0.1
SiO_2_	71.90	0.13
CaO	11.69	53.41
Na_2_O	13.00	0.68
Al_2_O_3_	1.39	-
MgO	1.43	0.18
K_2_O	0.15	-
P_2_O_5_	0.05	0.06
SO_3_	0.15	0.08
Fe_2_O_3_	0.15	0.04
ZnO	0.03	0.05
SrO	0.01	0.14
Others	0.05	45.23
Total	100	100

**Table 3:** IR absorption band and foam glass-ceramics band assignment for 1 and 6 wt% samples at various temperatures.

Wavenumber (cm^−1^)	Vibrational Mode Assignment
400–600	Ca-O stretching mode, O-Si-O bending mode
600–650	Ca-O-Si vibration band
800–1250	Si-O symmetric stretching mode, O-Si-O bending mode
1440–1450	C-O bond

## Data Availability

Data is contained within the article.
